# Non-Coding RNAs in the Transcriptional Network That Differentiates Skeletal Muscles of Sedentary from Long-Term Endurance- and Resistance-Trained Elderly

**DOI:** 10.3390/ijms22041539

**Published:** 2021-02-03

**Authors:** Paola De Sanctis, Giuseppe Filardo, Provvidenza Maria Abruzzo, Annalisa Astolfi, Alessandra Bolotta, Valentina Indio, Alessandro Di Martino, Christian Hofer, Helmut Kern, Stefan Löfler, Maurilio Marcacci, Marina Marini, Sandra Zampieri, Cinzia Zucchini

**Affiliations:** 1 Department of Experimental, Diagnostic and Specialty Medicine, University of Bologna School of Medicine, 40138 Bologna, Italy; paola.desanctis@unibo.it (P.D.S.); marina.marini@unibo.it (M.M.); cinzia.zucchini@unibo.it (C.Z.); 2 Applied and Translational Research Center, IRCCS Istituto Ortopedico Rizzoli, 40136 Bologna, Italy; g.filardo@biomec.ior.it; 3 Istituto di Ricovero e Cura a Carattere Scientifico (IRCCS), Fondazione Don Carlo Gnocchi, 20148 Milan, Italy; 4 Giorgio Prodi Interdepartimental Center for Cancer Research, S.Orsola-Malpighi Hospital, 40138 Bologna, Italy; annalisa.astolfi@unibo.it (A.A.); valentina.indio2@unibo.it (V.I.); 5 Department of Translational Medicine, University of Ferrara, 44121 Ferrara, Italy; 6 Second Orthopaedic and Traumatologic Clinic, IRCCS Istituto Ortopedico Rizzoli, 40136 Bologna, Italy; aledimartino75@gmail.com; 7 Ludwig Boltzmann Institute for Rehabilitation Research, 3100 St. Pölten, Austria; christian.hofer@rehabilitation.lbg.ac.at (C.H.); helmut@kern-reha.at (H.K.); stefan.loefler@rehabilitation.lbg.ac.at (S.L.); 8 Department of Biomedical Sciences, Humanitas University, Pieve Emanuele, 20090 Milan, Italy; maurilio.marcacci@humanitas.it; 9 Department of Surgery, Oncology and Gastroenterology, University of Padua, 35122 Padua, Italy; sanzamp@unipd.it; 10 Department of Biomedical Sciences, University of Padua, 35131 Padua, Italy

**Keywords:** skeletal muscle, aging, exercise training, gene expression, non-coding RNAs

## Abstract

In a previous study, the whole transcriptome of the vastus lateralis muscle from sedentary elderly and from age-matched athletes with an exceptional record of high-intensity, life-long exercise training was compared—the two groups representing the two extremes on a physical activity scale. Exercise training enabled the skeletal muscle to counteract age-related sarcopenia by inducing a wide range of adaptations, sustained by the expression of protein-coding genes involved in energy handling, proteostasis, cytoskeletal organization, inflammation control, and cellular senescence. Building on the previous study, we examined here the network of non-coding RNAs participating in the orchestration of gene expression and identified differentially expressed micro- and long-non-coding RNAs and some of their possible targets and roles. Unsupervised hierarchical clustering analyses of all non-coding RNAs were able to discriminate between sedentary and trained individuals, regardless of the exercise typology. Validated targets of differentially expressed miRNA were grouped by KEGG analysis, which pointed to functional areas involved in cell cycle, cytoskeletal control, longevity, and many signaling pathways, including AMP-activated protein kinase (AMPK) and mammalian target of rapamycin (mTOR), which had been shown to be pivotal in the modulation of the effects of high-intensity, life-long exercise training. The analysis of differentially expressed long-non-coding RNAs identified transcriptional networks, involving lncRNAs, miRNAs and mRNAs, affecting processes in line with the beneficial role of exercise training.

## 1. Introduction

Sarcopenia is one of the most common ailments of older age and the main cause for frailty. It is characterized by the progressive loss of muscle mass and strength and progressively leads to increased risk of falls and of permanent disability [1]. Inactivity is probably the major cause of muscle atrophy and the consequent oxidative stress links age-related sarcopenia to the sarcopenia due to muscle unloading, denervation and inflammation [2,3,4]. Therefore, physical activity is deemed to be the best countermeasure to aging-related sarcopenia [5]. Although most Authors agree on the fact that resistance training is the most effective intervention for increasing muscle mass and subsequently improving muscle function, a program, including a combination of resistance, endurance, balance and flexibility training may be best fit to reduce, delay, or even reverse sarcopenia and frailty in the elderly [6].

We recently provided evidence of the fact that life-long high-level exercise training can offset most pathways leading to age-related sarcopenia by counteracting mitochondrial dysfunction, inflammation, cellular senescence, impairment of proteostatic mechanisms, and fostering an efficient use of energy. All these mechanisms were studied at the level of protein-coding gene expression and were found to be remarkably independent of the type of exercise training (endurance or resistance, ET or RT) [7]. The study took advantage of the availability of vastus lateralis (VL) biopsies from senior exceptional amateur athletes (65–79 years of age) with a life-long practice of high-level physical activity [8], which were compared to biopsies from age-matched sedentary individuals. Protein-coding gene expression was evaluated in light of the results obtained by Next Generation Sequence (NGS) analysis and relevant gene functions were examined with the aid of the Kyoto Encyclopedia of Genes and Genomes (KEGG).

Although protein synthesis and, consequently, cellular composition and activity, ultimately depend on the expression of protein-coding genes, in the last twenty years the concept has been established that the expression of protein-coding genes is in turn dependent on a complex transcriptional network, including a multiplicity of non-coding RNAs [9,10,11,12,13]. While the Human Genome Project concluded that protein-coding genes constitute only about 2% of the human genome (International Human Genome Sequencing Consortium, [14]), the results of the ENCyclopedia Of DNA Elements (ENCODE) project revealed that almost the whole genome is involved in transcription, in particular, 80.4% of the genome is functional in at least one cell type (The ENCODE project Consortium, [15]). Non-coding RNAs (ncRNA) constitute a broad category which is involved in such diverse functions as translation, RNA splicing, DNA replication and gene regulation.

In particular, microRNAs (miRNAs) are small (~22-nt) trans-acting functional RNAs involved in silencing and post-transcriptional regulation of gene expression [16]. They take advantage of base complementarity to base-pair with sequences within mRNA molecules. The process causes mRNA silencing by either decapping or accelerated shortening of its poly(A) tail or by decreased efficiency of its translation by ribosomes, or more rarely in bilaterian animals through cleavage of the mRNA strand. The number of miRNA is estimated to be 519 in human [17]. Most of them are evolutionary conserved, as a matter of fact, the first miRNA was discovered in the nematode *C. elegans*. According to Friedman et al. [18] over 64% of human mRNAs are regulated by at least one miRNA and the average number of conserved targets per miRNA family exceeds 400. Many algorithms have been elaborated to predict targets of specific miRNAs, taking advantage of the fact that target recognition is done mainly through base pairing between the “miRNA seed”, i.e., miRNA nucleotides 2–7, and regions within the 3′ UTRs of target mRNAs. However, there are exceptions as well as additional pairing regions and regulatory sites. Computational methods aimed at identifying potential miRNA targets should be accompanied by the experimental validation showing RNA or protein downregulation. Databases reporting both validated (MiRTarBase, [19]) and not-validated (TargetScan, [20], and Targetminer, [21]) targets are available. The regulation of miRNA expression, maturation and decay is controlled by multiple mechanisms, some of them involving lncRNAs [22].

A special class of miRNA that appears to be selectively or preferentially expressed in striated muscle has been dubbed “myomiR” (reviewed in [23]). There are eight “canonical” myomiRs (miR-1, miR-133a, miR-133b, miR-206, miR-208a, miR-208b, miR-486, miR-499), whose expression is controlled by muscle-associated transcription factors, so that they participate in myogenesis, together with other miRNAs that are not exclusively or preferentially expressed in muscular cells [23]. While most myomiRs are expressed both in the heart and skeletal muscle, miR-208a is cardio-specific, and miR-206 is skeletal muscle-specific [24]. A detailed review on the role of miRNAs in satellite cell renewal has been recently published, with special attention to the myogenic factors that are targeted by miRNAs at each differentiation step [25]. In addition, a recent review [26] reported a number of variations in myomiR expression as a function of physical exercise, including the upregulation of miR-1 and miR-133a following acute exercise, but their downregulation after chronic exercise. During the last decade, evidence that miRNA can also be found in body fluids, and specifically in plasma, started to accumulate. These “circulating miRNAs” are generally carried by membrane-bound vesicles, hence the name “exomiRs”; they can be a novel kind of cell-to-cell communication [27]. Due to their stability, they are being investigated as potential markers in different pathological and physiological situations, including the evaluation of the exercise status [28,29]. Since they may have a different composition from the intracellular miRNAs, and since our study was restricted to intracellular ncRNAs, circulating ncRNAs will not be further discussed here.

Another very abundant category of ncRNAs (~270,000 transcripts) consists of long-ncRNAs (lncRNAs), which are characterized by the fact that transcripts are 200 nt or longer. They share many features with mRNAs, including 5′ capping, splicing, and poly-adenylation, but most of them are not translated into proteins [30]. They are a highly heterogeneous, still poorly defined group, with a very high tissue- and developmental-stage specificity. Although it is generally believed that they are involved in gene regulation, only few of them have been functionally characterized and they do not exhibit high levels of conservation [31]. It is believed that some of their functions may depend more on their secondary structure than on their primary sequence. Their three-dimensional structures and the ability to interact with auxiliary factors enables them to interact with DNA, RNA and proteins, to sequester them or to hinder their binding sites in order to interfere with their function. Antisense RNAs, that is RNAs that are transcribed from the opposite DNA strand of a protein-coding gene, are sometimes classified within lncRNAs, although some of them are less than 200 nt long. They are involved in epigenetic mechanisms leading to transcriptional regulation and in post-transcriptional modulation [32]. Another class of lncRNAs, devoid of a poly-A tail, emerged from cancer studies, which led to the discovery of the so-called *small nucleolar RNA host genes* (*SNHGs*), which are reportedly overexpressed in various cancers. They appear to carry out a variety of roles, including interaction with methyltransferases and with transcription factors, prevention of protein ubiquitination and activity of miRNA sponging, which indirectly up-regulates the translation of miRNA targets [33,34]. The concept of miRNA sponging is part of the broader hypothesis of competitive endogenous RNA (ceRNA), which proposes that RNA molecules with shared miRNA binding sites, such as lncRNAs, different mRNAs, pseudogene transcripts, and circular RNAs, compete for miRNA binding, thus exerting a post-transcriptional control [35]. In short, lncRNAs can either repress or activate gene expression through a variety of mechanisms.

From this brief overview, the complexity of the ncRNA landscape clearly emerges, as well as the multiple interactions that occur between different types of RNA and between the RNAs and the machinery involved in the overall regulation of protein synthesis. We have recently compared the mRNA transcripts of VL from two groups of elderly, representing the two ends of a physical activity scale, the study revealed the intertwining of different pathways, in some cases conflicting, which contributed to the maintenance of a substantial efficiency of the skeletal muscle in the well-trained elderly [7]. The purpose of the present study is to analyze the miRNA and lncRNA espression profile in trained and sedentary elderly, going beyond the mRNA transcriptome, in order to shed light on the underlying transcriptional network in the skeletal muscle, aimed at finely tuning relevant activities involved in the maintenance of muscular performance in old age.

## 2. Results

### 2.1. Micro RNA

The Affimetrix arrays identified 6599 sequences from VL biopsies of trained (TRA) and sedentary (SED) subjects, including miRNA precursors and short RNAs not belonging to the miRNA family. Data are reported in Table S1 and have been deposited in GEO (GSE165633). By setting the statistical significance at Adj *p* Value < 0.05, no significant differences in their expression were found between subjects who practiced either endurance or resistance training (ET versus RT), in analogy with what we described for mRNA expression in our previous paper [7]. As a matter of fact, 71 out of a total 6599 sequences resulted to be differentially expressed in TRA versus SED subjects. Of them 32 were miRNAs (Table 1). Notably, among the eight “canonical” myomiRs, only miR-486 was differentially expressed between TRA and SED subjects, in particular both miR-486-3p and miR-486-5p were upregulated in TRA in comparison to SED, while other myomiRs were expressed in both groups, however, without statistically significant dif ferences between TRA and SED.

**Figure 1:**
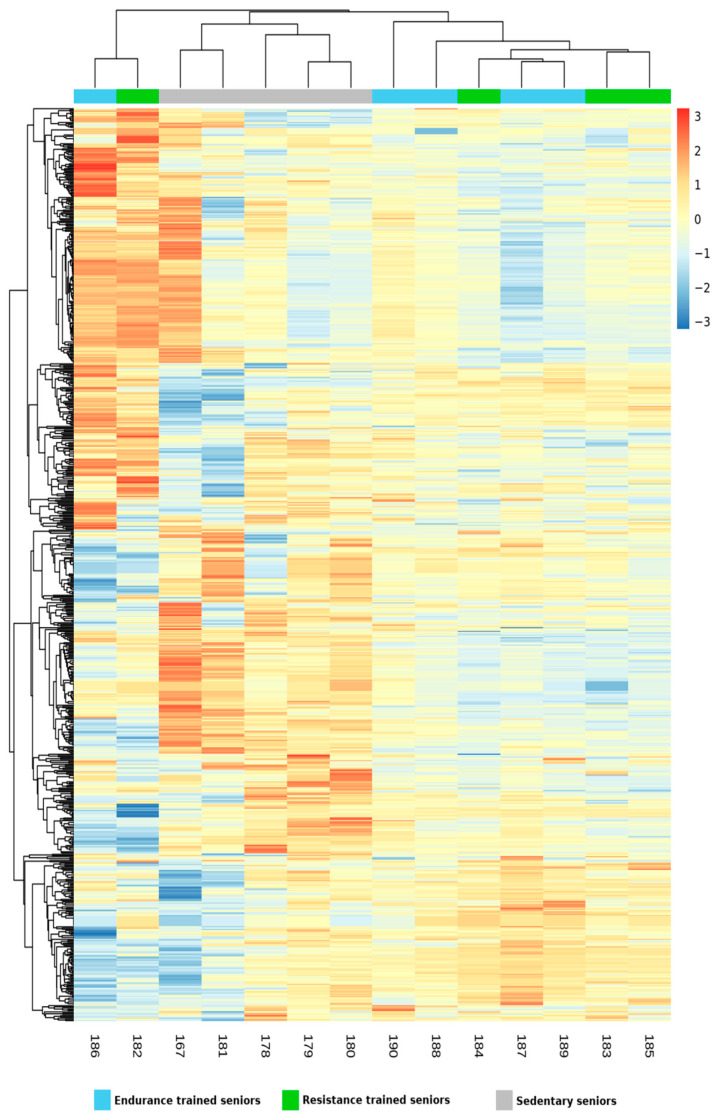
Heatmap generated by unsupervised hierarchical clustering analysis representing the expression data of miRNAs in endurance trained (ET), resistance trained (RT) and sedentary (SED) seniors. Each column corresponds to one subject while miRNAs are arranged along the rows. Color scale represents the mean centered log2 signal expression detected by the Affymetrix array, ranging from blue (less expressed) to red (more expressed). The hierarchical relationship between subjects and between miRNAs are represented in the form of dendograms in columns and in rows, respectively.

**Figure 2:**
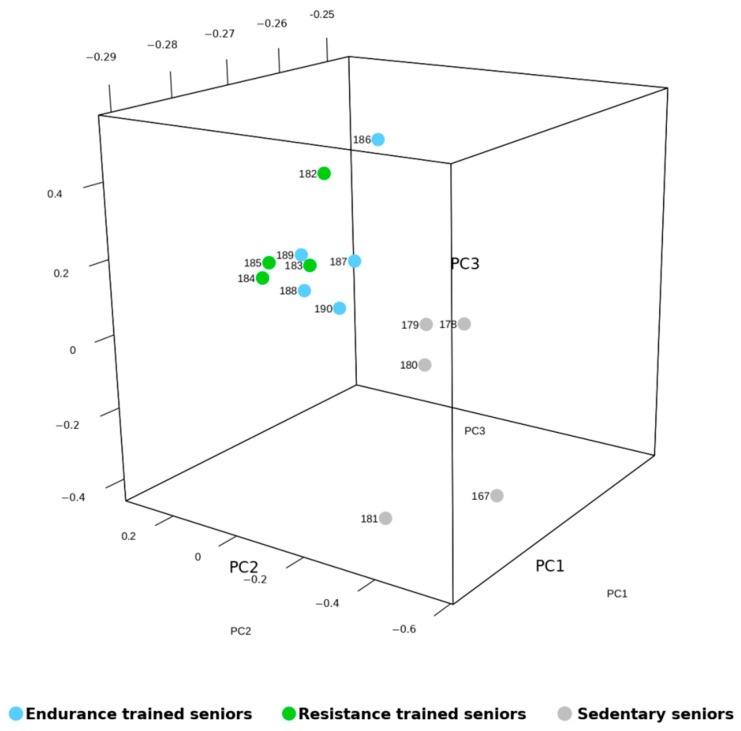
Principal Component Analysis (PCA) of miRNA expression data from trained (TRA) and sedentary (SED) vastus lateralis (VL). The plot shows the three-dimensional projections of the first 3 components generated by PCA. Each dot represents one subject. The higher the distance between dots the higher the difference of miRNA expression patterns; the distance from the dots of the different groups is proportional to their variance.

All identified miRNAs were then evaluated by mixture distribution analysis, in order to assess the similarities in miRNA expression shared by the three groups of elders, differing for amount and quality of physical activity. The heat map generated by the analysis, shown in Figure 1, grouped together two trained subjects, one RT and one ET, which bore some similarity also with the miRNA expression of one SED subject. However, the five SED subjects were correctly clustered together, while the remaining seven TRA subjects were separated from the SED ones and formed a separate cluster. In such a way, the unsupervised hyerarchical clustering analysis confirmed the results obtained by the statistical evaluation of miRNA expression, but also highlighted the fact that two samples, namely ET 182 and RT 186, had a distinct miRNA expression pattern. In order to understand what made these two senior athletes different from the other TRA subjects and so similar to each other, we re-evaluated their anthropometric features and their training routines. They did not share any peculiarity in such features, such as Body Mass Index (BMI) or training level, none of them practiced game sports, ET 182 training was 87.5% endurance and 12.5% resistance, whereas RT 186 trained 100% in resistance mode. Notably, also the mRNA expression of ET 182 made him cluster “erroneously” within the group of RT athletes, together with another ET subject [7]. In any case, we confirmed the fact that SED miRNA profile could be distinguished from that of TRA subjects also by Principal Component Analysis (PCA) (Figure 2), which grouped TRA and SED in two distinguished clusters along the PCA2 axis.

The list of validated mRNAs targeted by the 32 differentially expressed miRNAs was then obtained and compared to the list of differentially expressed mRNAs which had been the object of our previous investigation on the same VL samples [7]. Table 1 reports, for each differentially expressed miRNA, the number of experimentally validated targets according to the miRTarBase tool and the number of validated targets, which were at the same time differentially expressed in TRA and in SED and with opposite trends to their cognate miRNAs. These made up a panel of 989 genes, which were presumably modulated by miRNAs in the experimental context studied (Table S2). A considerable number of the differentially expressed miRNAs was found to target multiple mRNAs present in this panel, on the other hand, some mRNAs were the target of more than one miRNA. In particular, miR-20a-5p and miR-106-5p shared 108 mRNA targets, of which 19 belong to the pathways listed in Table 2.

We, then, evaluated this list of 989 mRNAs according to the KEGG, thus, grouping the mRNAs according to their function. The first 50 KEGG pathways are shown in Table S3 in decreasing order of combined score [36]. After excluding pathology-related pathways, the remaining 24 pathways (Table 2) were then compared to those characterizing the set of differentially expressed mRNAs, described in our previous manuscript [7], that highlight the relevant genes involved in the modulation of skeletal muscle morphological and functional adaptations to exercise training, and in counteracting sarcopenia. Remarkably, 6 KEGG pathways (in bold) overlapped with the highest scoring ones characterizing the set of differentially expressed mRNAs (AMPK signaling, Thyroid hormone; Focal adhesion, Cellular senescence, Insulin signaling, Ubiquitin-mediated proteolysis) [7], while a further 6 (in italic) were discussed in our mRNA manuscript as characterizing differentially regulated pathways or processes (Mitophagy, mTOR signaling, Autophagy, Adherens junction, Longevity regulating; regulation of actin cytoskeleton) [7]. This result shows that miRNA-operated post-translational regulation is especially relevant in genes modulated by exercise training in the elder.

The identification of the targets of the differentially expressed miRNAs leads to further interesting considerations. On one hand, this study may help to evaluate the global impact of miRNA-operated modulation on the regulation of gene expression, at least in the context of the adaptation of skeletal muscle to aging and to activity. It appears that only a minority of the genes that are modulated by exercise training is controlled by miRNAs: if one takes into consideration the relevant highest scoring KEGG shown in Table 2, the miRNA-regulated mRNAs were about 50 % or less, as far as can be deduced from the reliability of the Affimetrix data and from the current knowledge regarding the targets of miRNAs. For instance, the insulin pathway includes 137 genes, of which 84 were differentially expressed in TRA versus SED [7]. Only 18 of these genes (i.e., 22%) were targeted by differentially expressed miRNAs.

Notably, the above-mentioned manuscript underlined that the concurrent AMPK- and mTOR- signaling pathways were both involved in the training-induced adaptations, independently of the exercise typology. It is interesting to note that these same signaling pathways result from the functional analysis of miRNA-regulated genes. On the other hand, many targets of significantly modulated miRNAs are functionally classified within pathways that are not found among the first relevant ones identified in the previous analysis of differentially expressed mRNAs, for instance, miRNA-497 modulates nicotinamide nucleotide transhydrogenase (*NNT*), a gene involved in the regulation of the NAD+/NADH ratio [7]. These results pinpoint the fact that adaptation to exercise training involves the post-translational control of several cellular processes and of many signaling pathways, in particular, miRNA-regulated pathways involve also processes as cell cycle, ferroptosis, mRNA surveillance as well as TGF-beta, p53, FoxO, Hippo, WNT, MAPK, neurotrophin, sphingolipid and HIF-1 signaling pathways, which include differentially expressed genes not discussed in our previous paper.

**Figure 3:**
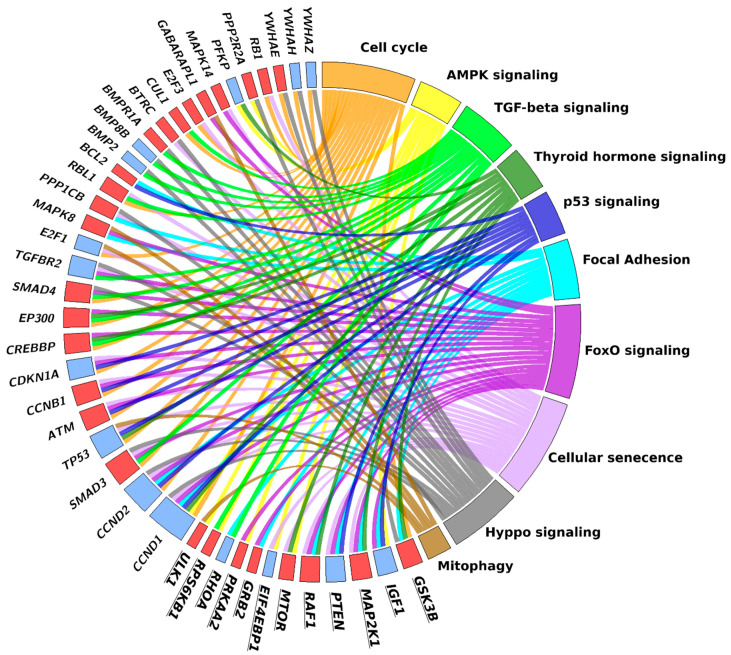
Chord plot showing the genes shared by at least 2 out of the first 10 pathways resulting from KEGG analysis of miRNA-regulated genes. The rectangle flanking the gene name is red when the gene is overexpressed in trained (TRA) versus sedentary (SED) vastus lateralis (VL), blue when it is underexpressed. The plot includes twelve genes (bold characters) of the mTOR pathway (the 11° pathway), which were grouped together to emphasize the relationships of this pathway with the others.

As already underlined when discussing the results of KEGG analysis for the differentially expressed mRNAs, a number of genes belonged to more than one pathway, thus showing that the involved pathways are highly interlaced. A substantial number of miRNA-regulated genes can be also attributed to more than one pathway, as shown in Figure 3, depicting miRNA-regulated genes common to at least 2 out of the first 10 pathways resulting from KEGG analysis.

Besides classifying the 989 genes targeted by the differentially expressed miRNAs according to the KEGG pathways, which confirmed the cause-effect relationship between targeted genes and contrast to sarcopenia, we extensively searched PubMed for further functional properties of the 32 differentially expressed miRNAs in the context of skeletal muscle. This led us to realize that, as we will discuss for lncRNAs, the majority of manuscripts on miRNAs are about cancer. Further information about some of the 32 differentially expressed miRNA was nevertheless gathered and is reported below.

- **miR-181a-2-3p** (upregulated in TRA versus SED), already mentioned above, has an anti-inflammatory role [37].
- **miR-199a-5p** (downregulated in TRA versus SED) is modulated in the diabetic muscle, where its role in targeting Slc2a4/GLUT4 and Hk2/HK2 expression was confirmed [38], it is involved in myogenesis and dysregulated in Duchenne Muscular Dystrophy (DMD) [39], its upregulation, along with that of **miR-497-5p** and of other four miRNAs is associated with mitochondrial dysfunction in DMD [40].
- **miR-193a-3p** (downregulated in TRA versus SED) was found to be deregulated in Myotonic Dystrophy Type-2 [41].
- **miR-20a-5p** (upregulated in TRA versus SED) is involved in myoblast proliferation and differentiation [42].
- **miR-486-5p** (upregulated in TRA versus SED), the only myomiR differentially expressed in TRA versus SED VL samples, is rightly considered a myogenesis regulator, since it is implicated in the inhibition of myocardin-related transcription factor A (MRTF-A), which modulates one of the last steps of myogenic differentiation [43]; interestingly, it can be upregulated by administration of green tea polyphenol epigallocatechin-3-gallate, with benefits for the ageing skeletal muscle [44]; moreover, its involvement in a complex metabolic regulation of muscle hypertrophy during aerobic exercise was reported [45].
- **miR-100-5p** (downregulated in TRA versus SED) is involved in the same complex metabolic regulation following aerobic exercise that has been reported for miR-486-5p [45].

### 2.2. Long Non-Coding RNA

**Figure 4:**
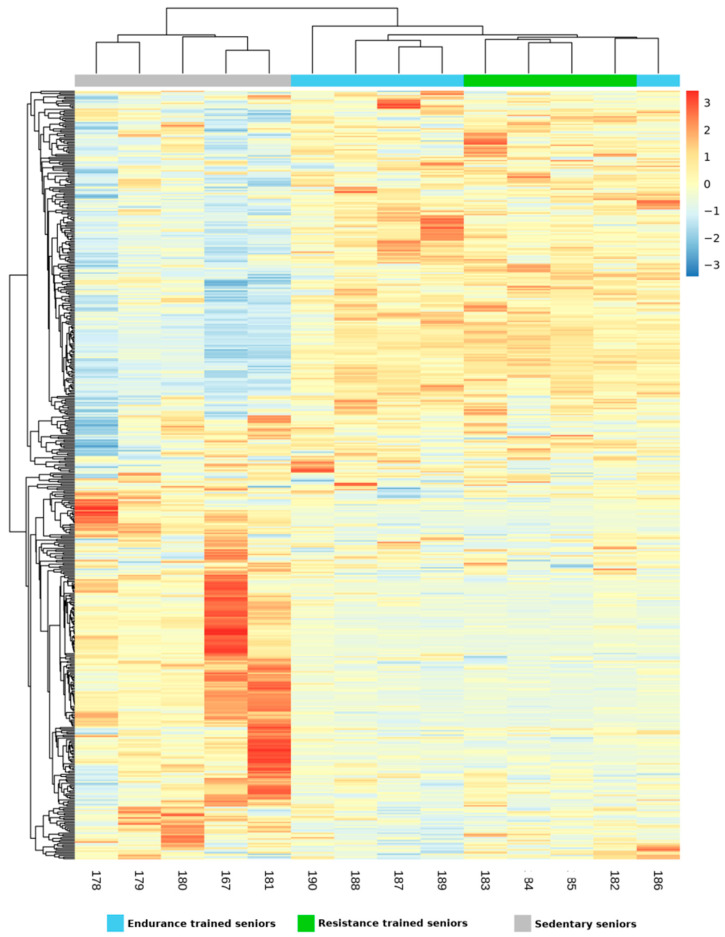
Heatmap generated by unsupervised hierarchical clustering analysis representing the expression data of lncRNAs in endurance trained (ET), resistance trained (RT) and sedentary (SED) seniors. Each column corresponds to one subject while lncRNAs are arranged along the rows. Color scale represents the mean centered log2 signal expression detected by the Affymetrix array, ranging from blue (less expressed) to red (more expressed). The hierarchical relationship between subjects and between lncRNAs are represented in the form of dendograms in columns and in rows, respectively.

**Figure 5:**
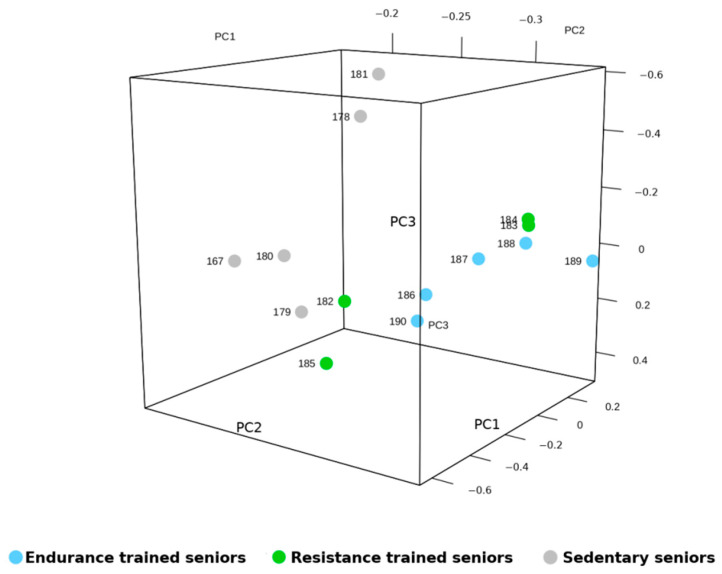
Principal Component Analysis (PCA) of lncRNA expression data from trained (TRA) and sedentary (SED) vastus lateralis (VL). The plot shows the three-dimensional projections of the first 3 components generated by PCA. Each dot represents one subject. The higher the distance between dots the higher the difference of lncRNA expression patterns; the distance from the dots of the different groups is proportional to their variance.

Data obtained by NGS analysis have been deposited in GEO (GSE165633). After filtering for those samples that did not reach at least 3 counts per million (cpm) in at least 2 samples, a total of 511 lncRNAs were identified by NGS analysis in VL biopsies of trained and sedentary elder subjects (Table S4). By setting the statistical significance at Adj *p* Value < 0.05, significant expression differences between TRA or SED subjects were found in 242 lncRNAs, whereas ET and RT subjects did not differ on this respect. Mixture distribution analysis was performed on the expressed lncRNAs in the same way as it was done for miRNAs, to evaluate similarities and differences in lncRNA expression across the three groups of elders. A heatmap was thus generated (Figure 4), where the TRA group clustered in a clearly distinct way from the SED group. Within the TRA group, four out of five ET clustered together, while ET 186 clustered with RT subjects, adjacent to RT 182, confirming a strong similarity within ET 186 and RT 182, as observed with miRNA expression. The PCA (Figure 5) grouped SED subjects on one side along the PC2 axis, but RT 182 and RT 185 were localized very close to SED, the remaining TRA subjects were mixed together. As a whole, these two analyses confirmed the distinct expression pattern of SED and TRA subjects, and the fact that ET and RT shared many common features.

The heterogeneity of lncRNAs as a category is reflected in the different classes of RNAs that were identified. A large number of RNAs were uncharacterized, some were transcribed pseudogenes and the majority belonged to one of the following categories: long non-protein coding RNAs, whether intergenic or not, antisense lncRNAs, small nucleolar RNA host genes. When a target was identified by LncRNA2target database, either the mRNA list retrieved in the previous work or the miRNA list obtained in the present one were searched and, if the target was differentially expressed, its concordance with the lncRNA was evaluated. According to the different roles played by the lncRNAs towards interacting miRNAs and mRNAs, the lncRNA-target pair could be found bearing the same “sign” or the opposite one, i.e., both members could be upregulated or downregulated in TRA subjects with respect to SED ones, or one member could be up- and the other down-regulated, implying the complexity of the relationship of repression/inhibition of one member of the pair towards the other. For instance, lncRNA *GAS5* targets 12 mRNAs and 1 miRNA present in our lists of differentially expressed genes (Table 3). *GAS5* was dowregulated in TRA compared to SED; 8 of its target mRNAs were downregulated as well, whereas 4 mRNAs and the miRNA were overexpressed.

**Figure 6:**
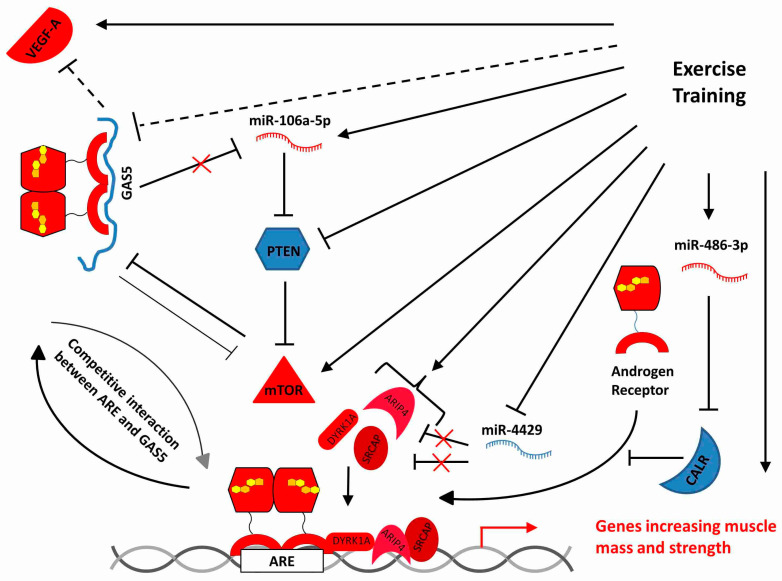
A simplified transcriptional network illustrating hypothesized interactions involving one lncRNA, three miRNAs and some protein-coding genes in the vastus lateralis of senior sportmen. Solid lines are used here to indicate direct relationships of activation or inhibition. Dashed lines are used here to indicate relationships that may be mediated by other factors. Proteins are shown in place of their mRNAs. RNAs and proteins are colored blue to indicate downregulation, red for upregulation.

As an example of the complex interactions involving messenger, long-non-coding and micro RNAs, Figure 6 shows a hypothetical view of the transcriptional network regulating the transactivating activity of the androgen hormone receptor, involving lnc-RNA *GAS5*, miR-106a-5p, miR-486-3p, miR-4429, and several protein-coding genes, based on expression data presented here and in [7] and on data found in [46,47,48,49]. As discussed by Wyce et al. [47], the androgen receptor has a critical role in muscle function, a circumstance which makes this example a particularly relevant one. In fact, we showed that life-long, high-intensity exercise training activates the mTOR pathway and upregulates several, but not all, genes downstream of the androgen receptor signaling, that are involved in increasing muscle mass and strength: *ACTA1*, *MYOM1*, *MYOM2*, *MYOM3*, *MYOT*, *MEF2C*, *SMYD1*, as well as the major angiogenetic factor *VEGFA* and the mTOR pathway; on the other hand, it downregulates *CALR* and *PTEN*. Moreover, exercise training upregulates miR-106a-5p and miR-486-3p and downregulates miR-4429 and by mechanisms still unknown in detail, lnc-RNA *GAS5*. *GAS5* may bind, by decoy affinity, the androgen receptor, thus attenuating the androgen receptor response. It also inhibits *VEGFA* and “sponges” miR-106a-5p. In the present scheme, depicting a hypothesized detail in the gene expression network occurring in the skeletal muscle of well-trained elders, *GAS5* is unable to exert such inhibitory activities since it is downregulated. *GAS5* downregulation is mediated, among other mechanisms, by the transcriptional repression operated by mTOR, which, in turn, could be negatively controlled by *PTEN* (downregulated here), and, indirectly, protected by miR-106a-5p. The transcriptional activation of miR-486-3p inhibits the expression of *CALR*, which is thus unable to repress the binding of the androgen receptor to its DNA binding site (ARE, Androgen Response Element). *GAS5* competes with the DNA response elements for the binding with any steroid receptor (androgen, progesteron, mineralcorticoid, glucocorticoid). The transcription at the ARE binding site requires a number of co-trascriptional activators, including DYRK1A, ARIP4, and SRCAP; the mRNAs coding for these cofactors are upregulated in TRA VL, while *ARIP4* and *SRCAP* mRNAs are targeted by miR-4429, which is downregulated in TRA VL.

Only a few of the 242 differentially expressed lncRNAs have been the object of scientific publications. Information on those we deemed as the more interesting ones for the model studied here is shown in Table 3 and briefly summarized below.

- *Small Nucleolar RNA Host Gene G7* (***SNHG7***). Upon observing a positive correlation between *SNHG7* and *GALNT7* and a negative correlation between *SNHG7* and miR-34a in colorectal cancer lines. it was suggested that *SNHG7* upregulates *GALNT7* expression by sponging miR-34a [50]. Another paper reported that *SNHG7* antagonizes miR-193b to increase the expression of *FAIM2* [51]. By evaluating our data sets, we confirmed the positive correlation between *SNHG7* and both *GALNT7* and *FAIM2*. since the three appear to be significantly downregulated in TRA in comparison with SED samples, on the other hand, neither miR-34a or miR-193b were differentially expressed. While the effect of *GALNT7* and *FAIM2* in tumor progression is known (the former favoring and the latter impairing tumor growth), their function in the context of skeletal muscle, exercise and contrast to sarcopenia is less obvious. *GALNT7* is involved in several transcriptional networks, including the one involving the competing endogenous *RNA TP73-AS1*—which sequesters miR-103a, and *RP11-798M19.6*. There is evidence that *FAIM2* expression may increase susceptibility to type 2 diabetes associated with obesity [52] and to obstructive sleep apnea-related cardiac injury [53], thus, the *FAIM2* downregulation (found in TRA subjects) appears to bear favorable effects.
- *Growth Arrest Specific 5* (non-protein coding) (***GAS5***) is a small nucleolar RNA host gene, acting as a tumor suppressor, probably by favoring apoptosis [54]. It has multiple interactions with miRNAs [48] and interacts with mTOR in a complex reciprocal inhibitory relationship [55]. Interestingly, part of the secondary RNA structure of the encoded transcript mimics the glucocorticoid response element, thus interfering with its binding with the glucocorticoid receptor, as well as with the binding of androgen, progesterone and mineralocorticoid receptors to their response elements [48,56]. *GAS5* is downregulated in TRA VL, thus favoring the activation of the above mentioned receptors, with particular emphasis on the anti-inflammatory role of glucocorticoid receptor transactivation and the role of androgens in increasing muscle mass and strength. *GAS5* interacts with multiple mRNAs and miRNAs, in particular it is targeted by miR-20a-5p and miR-106, both upregulated in TRA VL. Moreover, it was shown that *GAS5* knockdown upregulates *VEGFA* [57], an angiogenetic growth factor which is overexpressed in TRA skeletal muscles.
- *Small Nucleolar RNA host gene 12* (***SNHG12***) acts as an oncogene, by sponging miR-181a [58]. Soriano-Arroquia et al. [59] identified miR-181a as a positive regulator of the sirtuin1 (*SIRT1*) gene expression in skeletal muscle and the miR-181a:*SIRT1* interactions as regulators of myotube size. They also reported that the expression of miR-181a and of *SIRT1* was decreased in skeletal muscle from old mice. In another paper published in 2020, miR-181a was identified as putative regulator of mitochondrial dynamics [60]. Therefore, the fact that we report here a downregulation of *SNHG12* coupled with an upregulation of its target miR-181a in TRA VL constitutes a further advancement in the understanding of the mechanisms underlying the preservation of the mitochondrial function and of skeletal muscle morphology and performance induced by long-term high-level training in elders [7].
- *HOXA Transcript Antisense RNA, Myeloid-specific 1* (***HOTAIRM1***) is one of the best studied lncRNA, because of its possible oncogenic role [61]. In TRA VL *HOTAIRM1* is downregulated and one of its targets, miR-20a-5p is upregulated. It is difficult to realize the role of the pair *HOTAIM1*-miR-20a-5p in the specific context of skeletal muscle, exercise training and contrast to sarcopenia since there are 190 genes that are both validated targets of miR-20a-5p and significantly downregulated in TRA VL. The specific functions of these 190 gene products range from being part of signaling pathways, regulating cell cycle and apoptosis, controlling transcription and translation, including the synthesis of a number of ribosomal proteins, as pointed out above.
- *Metastasis associated lung adenocarcinoma transcript 1* (***MALAT1***) is one of the best studied lncRNA. It promotes epithelial-mesenchymal transition by activating both the TGF-beta and the WNT pathways, as well as angiogenesis. Reciprocal repression between *MALAT1* and miR-140 was demonstrated in human gliomas [62]. Liu et al. [63] reported that exercise downregulated *MALAT1* expression in the insulin-resistant mouse model, resulting in reduced resistin levels. Neither study apparently applies to our experimental setting, since *MALAT1* was upregulated in TRA versus SED VL, but miR-140 was not differentially expressed; therefore, the effect of exercise training in *MALAT1* regulation is apparently different in subjects that are not insulin resistant.
- Other differentially expressed lncRNAs are involved in tumorigenesis. Summarizing, ***SNHG7***, ***SNHG12***, ***HOTAIRM1***, ***SNHG14***, ***HOXD-AS1***, ***SNHG15***, ***SNHG1***, ***LINC00152***, ***HOXC-AS1***,***MALAT1*** have all been described to promote cell proliferation, invasion and migration, though by different mechanisms. Apart from *SNHG14* and *MALAT1*, they were all downregulated in TRA VL. On the other hand, ***GAS5*** and ***TP73-AS1*** have tumor suppressor activities, the former being down- and the latter up-regulated in TRA VL. The relationships of these lncRNAs with the experimental model, presented here, is difficult to interpret.

## 3. Discussion

All cellular properties and activities depend on the quality and quantity of the expressed proteins, which change in a specific way according to the changing needs and conditions of tissues and organisms. The modulation of protein expression is achieved through the regulation of protein-coding gene expression, which in turn, depends on both transcriptional and post-transcriptional mechanisms. Skeletal muscle is an interesting model for studying the modulation of gene expression, since it easily undergoes rapid adaptations according to the activity level, nutrition, aging, and other conditions. Therefore, a full understanding of the forces that oversee changes related to aging and/or training in skeletal muscle requires an investigation that examines both the transcriptional and the post-transcriptional levels.

We compared the gene expression of biopsies from the leg muscle VL of an exceptional group of aged men who had trained at high level for at least 30 years and that of sedentary age-matched controls. While mRNA expression was described and thoroughly discussed in an earlier manuscript [7], this study takes into consideration micro- and long-non-coding RNAs. Genome-wide approaches enabled us to recognize all expressed RNA sequences: in fact, the Affimetrix arrays identified 6599 short sequences and the NGS identified 511 lncRNAs.

From the 6599 short RNA sequences, only 32 were differentially expressed miRNAs (i.e., differed in TRA and SED subjects). We, then, tried to understand their role in gene modulation by identifying their validated targets and looking for differentially expressed mRNA targets that had a trend opposite to that of their cognate miRNA. By analyzing the 989 genes thus identified by the KEGG, it was possible to gather interesting insights. Nevertheless, we are aware of the fact that in such a way we limited our study to only one of the main mechanisms of miRNA-mediated gene expression silencing—the promotion of mRNA decay. The other main mechanism by which miRNAs regulate gene expression—translational repression—requires that transcriptomics be accompanied by proteomics. This is one of the main limitations of the present study.

As for lncRNAs, 242 out of 511 expressed sequences were differentially expressed in TRA and SED VL biopsies. The attempt to assign a role to these lncRNAs in the molecular mechanisms allowing exercise training to counteract the aging-related loss of muscle mass and function encountered a major limitation of a different kind. In fact, lncRNA functions have been extensively studied only in the context of cancer, while studies in different pathological and physiological contexts are scanty. Moreover, the lncRNA field is still rapidly evolving, and little is known about the majority of the expressed lncRNAs.

Unlike lncRNAs, miRNAs have been rather extensively studied in the context of skeletal muscle, aging, and exercise. As we mentioned above, muscle-specific miRNAs, myomiRs, have a recognized role in myogenesis [23], but their role in the adaptation to exercise is complex [26,64]. In our samples, miR-486 was the only myomiR differentially expressed in TRA versus SED. In an omic- approach to understand the roles of miRNAs as biomarkers of skeletal muscle strength, Mitchell et al. [65] identified miR-486-5p, predicted to target the anabolic PI3K/Akt signaling, as the most highly expressed miRNA; other 3 miRNAs (miR-10b-5p, miR-133a-3p, and miR-22-3p) were highly expressed; only 486-5p was however upregulated in TRA samples studied in the present work. In a study examining miRNA changes in skeletal muscle ageing, Soriano-Arroquia et al. [59], found that 16 miRNAs were downregulated and 14 upregulated in ageing muscles; of these, only miR-181a was differentially expressed in the VL samples studied here (downregulated in ageing muscles and upregulated in trained ones). Similar discrepancies were found with several other literature reports [66,67,68,69,70,71,72]. Collectively speaking, these studies examining the miRNA expression in exercise training and/or in ageing were carried out on subjects who were exposed to a relatively short-term training. This might explain the discrepancies we observed with our results, since the trained elders of our experimental model had a very elevated level of training, in terms of intensity and duration in time, which allowed them to really counteract age-related decline in muscle mass and strength [7].

As specified in the Result section, functional analysis of miRNA-targeted genes was able to confirm the involvement of miRNAs in pathways modulating skeletal muscle morphological and functional adaptations to exercise training. About half of the highest-ranking KEGG pathways grouping miRNA-targeted genes had been already described within the transcriptomic analysis of protein-coding genes that were modulated by long-term high-level exercise training in the skeletal muscle of elder men, while the other pathways and processes are a proof of the complexity of the genetic modulation exerted by long-term high-level training in the skeletal muscle. Among the signaling pathways grouping miRNA-targeted genes, however, the presence of the, “Neurotrophin signaling pathway”, was somehow unexpected, since one would expect neurotrophins to be expressed in the nervous tissue, where they exert beneficial tasks. This finding may reflect the fact that muscle fibers of physically active seniors experience frequent re-innervation processes [8], but another explanation may coexist with the former: A recent paper [73] reported that the plasma of exercise-trained rats was enriched with proteins of the neurotrophin signaling pathway. Therefore, it may be hypothesized that the exercised skeletal muscle is one of the site of synthesis of these proteins, which are then released to serve in skeletal muscle-to-nervous system long-distance communication, with beneficial effects in the protection against neurological and mental disorders, and in improving memory and learning, particularly in the elders [74,75].

Interestingly enough, the present study underlines the fact that only within a frame where the miRNA-operated repression of mRNAs is taken into consideration, one may understand some regulative processes outlined in [7]. In the skeletal muscle context, this may help to unravel the relationships between the two concurrent pathways mTOR and AMPK, *which are both upregulated in TRA subjects with respect to SED ones* [7]. In fact, we previously reported at least two somewhat paradoxical findings: (i) the mTOR pathway positively controls the expression of genes involved in ribosome biogenesis. However, these genes were dowregulated in TRA elders [7]. These data may now be explained through the upregulation of miR-20a-5p which targets most downregulated genes of the ribosome pathway; (ii) AMPK inhibits the protein biosynthesis process; one key component of the AMPK pathway is EIF4EBP1, which, by interacting with the eukaryotic initiation factor eIF4E, represses translation. Despite the overall activation of the AMPK pathway in TRA elders, *EIF4EBP1* mRNA was downregulated [7], thus, releasing the mRNA translation: the downregulation of *EIF4EBP1* may now be explained by the finding that it is targeted by miR-7847-3p, which is upregulated in TRA VL.

The analysis of differentially expressed lncRNAs was heavily hampered by the fact that many sequences have not yet been studied or have been examined only in the context of cancer and only allowed some speculation about the transcriptomic networks made up by mRNAs, miRNAs, and lncRNAs. A tentative scheme involving the regulation of the steroid receptor (i.e., androgen, progesteron, glucocorticoid, or mineralcorticoid receptor) by the interaction of different classes of RNAs has been depicted in Figure 6. A recent meta-analysis of skeletal muscle transcriptome [76] identified NR4C3, a member of the steroid-thyroid hormone-retinoid receptor superfamily, as one of the most exercise- and inactivity-responsive genes. Interestingly, *NR4C3*, which is highly overexpressed in TRA versus SED VL (Log2 Fc = 4.43) might well be the receptor involved in the transcriptional circuit tentatively sketched in Figure 6.

In order to validate transcriptional networks such as these, in vitro studies involving the genetic manipulation of the various components will be required. In general, larger studies, involving a higher number of subjects stratified for age and training level, may be necessary to better understand the role of ncRNAs in the transcriptional network modulating the pathways that counteract aging-associated sarcopenia.

## 4. Materials and Methods

### 4.1. Subjects, Biopsies and RNA Extraction

The RNA used in the present study was the same utilized for the transcriptomics study published earlier in 2020 by our group [7]. Shortly, the study was carried out on five control sedentary and nine highly-trained subjects, five of the latter were predominantly endurance trained and four were predominantly resistance trained. All subjects were male and between 65 and 79 years of age. All details about their anthropometric features, of their training routine and activity level and on force assessment can be found in the referenced paper. It is important to stress that all subjects were healthy, did not take any dietary supplement and that the trained subjects had indeed an exceptional level of training, as they had been training at least three times a week for at least 30 years. All participants were informed on the purposes of the study and the details of the biopsy and signed an informed consent form. The study on sedentary subjects was approved by the Ethical Committee of the IRCCS Istituto Ortopedico Rizzoli (Prot. gen. n.ro 0002659, 2012) and by the Ethical Committee of the City of Vienna (EK-08-102-0608, 2008) as for trained subjects. Vastus lateralis biopsies were taken about 20 cm above the knee. RNA was extracted and quality controlled by standard procedures as specified in the referenced manuscript.

### 4.2. RNA Analysis

The RNA samples were tailed with Poly(A) and labeled with biotin using the FlashTag™ Biotin HSR RNA Labeling Kit (Affymetrix, Inc., Santa Clara, CA, USA) according to the manufacturer’s instructions. The labeled RNA samples were hybridized onto the Affymetrix Gene-Chip miRNA 4.0 array (Homo sapiens), washed and stained on fluidics station 450, and then scanned with a Scanner 3000 (all from Affymetrix, Inc.). A customized R-bioconductor pipeline was adopted to carry out the analysis of differential miRNA expression. Firstly, the raw data (CEL files) were processed by Robust Multichip Average method (package *oligo,* function *rma*) to perform background subtraction, quantile normalization at single probe level. Then, *lmFit* and *eBayes* functions (form *limma* package) were adopted respectively to fit the linear model and to calculate the moderate t-statistics with the aim to compare the miRNA expression profile of trained versus sedentary subjects. Downstream analysis was performed in the subset of miRNAs with log2signal > 3 in at least 2 samples. A miRNA was considered significantly modulated if the Benjamini-Hochberg adjusted *p*-value was ≤0.05.

NGS analysis was applied for the evaluation of lncRNA transcripts, along with mRNAs. Technical details of NGS analysis are reported in the referenced manuscript. Briefly, whole-transcriptome RNA libraries were prepared in accordance with Illumina’s TruSeq RNA Sample Prep v2 protocol (Illumina, San Diego, CA, USA). Poly(A)-RNA molecules from 500 ng of total RNA were purified using oligo-dT magnetic beads, then reverse transcribed into double-stranded cDNA fragments. The products were then amplified to create the final cDNA library, then sequenced by synthesis (SBS) technology. The gene expression was quantified using the python function Htseq-count and normalized as count per million (cpm) with the R-bioconductor package edgeR.

#### 4.2.1. Heatmaps and Principal Component Analysis

Both miRNA and lncRNA gene sets were studied by unsupervised hierarchical clustering methods. Only probes with Log2signal >3 in at least 2 samples were considered for miRNA, whereas for ncRNA only genes with count per million >3 in at least 2 samples were considered. The heatmaps were built by using the R-bioconductor package *pheatmap* (clustering distance: *correlation*, clustering method: *average*), thus yielding a cladogram based on pairwise similarity in miRNA/lcnRNA expression. Principal Component Analysis (*R package prcomp*) was also applied as another means to further explore the dataset variability and to create a low-dimensional sample representation showing the relative similarity between the studied subjects.

#### 4.2.2. Micro- and Long Non Coding-RNA Targets

For each differentially expressed miRNA, experimentally validated microRNA-target interactions were sought using miRTarBase http://miRTarBase.cuhk.edu.cn/ [19]. To discriminate the validated miRNA target genes actually modulated in our experimental model, we took advantage from our previous transcriptional study of VL biopsies from the same elderly subjects enrolled for the present analysis [7], which reports a list of the significantly modulated genes in response to a life-long high-level training practice (for details on gene list and their selection criteria see both Material and Methods and Results sections of the cited article). Subsequently, we selected target genes validated by miRTarBase that, according to our previous data, were both significantly modulated in VL biopsies and expressed consistently with the expression of the related miRNAs (i.e., upregulated miRNA having a downregulated target gene or inversely). This screening yielded 989 genes, who were then evaluated by the KEGG (Release 91.0, 1 July 2019) using Enrichr, an integrative web-based software (https://bmcbioinformatics.biomedcentral.com/articles/10.1186/1471-2105-14-128) to elucidate their relevant functions. Fisher’s exact test *p*-values were calculated to identify which functional gene groups were significantly enriched. Graphical representation of enriched terms and the corresponding genes originated a chord plot, which was built with the software package Circos.

For each differentially expressed lncRNA, the identification of experimentally validated targets was attempted using LncRNA2Target v2.0, a comprehensive dataset of lncRNA-Target relationships identified by reviewing all published lncRNA papers (freely accessible at http://123.59.132.21/lncrna2target).

## Figures and Tables

**Table 1:** MicroRNAs differentially expressed in trained (TRA) vs sedentary (SED).

miRNA	Up- or Down-Regulated in TRA versus SED VL	logFC	*p* Value	Adj. *p*. Value	No. of Validated Targets	No. of Validated Targets That Are Differentially Expressed [7] and with a Trend Opposite to the Trensd of miRNAs
**hsa-miR-7847-3p**	Up	4.44	1.52 × 10^−8^	1.00 × 10^−4^	200	38
**hsa-let-7c-5p**	Down	−0.99	9.24 × 10^−8^	3.05 × 10^−4^	484	147
**hsa-miR-4298**	Up	5.02	1.45 × 10^−7^	3.18 × 10^−4^	46	7
**hsa-miR-6812-5p**	Up	2.46	5.92 × 10^−7^	9.77 × 10^−4^	65	17
**hsa-mir-3175**	Down	−3.51	2.12 × 10^−6^	1.75 × 10^−3^	205	53
**hsa-miR-3197**	Down	−3.10	1.98 × 10^−6^	1.75 × 10^−3^	23	4
**hsa-miR-3911**	Up	4.06	1.96 × 10^−6^	1.75 × 10^−3^	70	13
**hsa-miR-6510-5p**	Down	−1.47	5.31 × 10^−6^	2.69 × 10^−3^	85	21
**hsa-miR-4521**	Up	3.69	6.49 × 10^−6^	3.06 × 10^−3^	26	3
**hsa-miR-574-5p**	Down	−2.18	1.72 × 10^−5^	5.96 × 10^−3^	348	89
**hsa-miR-181a-2-3p**	Up	1.90	2.39 × 10^−5^	7.00 × 10^−3^	70	10
**hsa-miR-664b-3p**	Down	−0.86	2.94 × 10^−5^	7.46 × 10^−3^	145	43
**hsa-miR-199a-5p**	Down	−1.71	3.65 × 10^−5^	8.60 × 10^−3^	139	36
**hsa-miR-193a-3p**	Down	−2.56	3.65 × 10^−5^	8.60 × 10^−3^	115	41
**hsa-miR-4669**	Up	2.52	4.65 × 10^−5^	9.90 × 10^−3^	29	10
**hsa-miR-4269**	Down	−1.80	5.38 × 10^−5^	1.00 × 10^−2^	114	30
**hsa-miR-497-5p**	Down	−1.60	1.62 × 10^−4^	2.00 × 10^−2^	426	135
**hsa-miR-4485-3p**	Up	2.00	1.60 × 10^−4^	2.55 × 10^−2^	6	1
**hsa-miR-7110-5p**	Down	−3.47	1.66 × 10^−4^	2.55 × 10^−2^	87	20
**hsa-miR-5100**	Down	−1.43	2.01 × 10^−4^	2.82 × 10^−2^	57	19
**hsa-miR-486-3p **	Up	1.72	2.17 × 10^−4^	2.98 × 10^−2^	133	21
**hsa-miR-7150**	Down	−2.68	2.31 × 10^−4^	3.11 × 10^−2^	102	28
**hsa-miR-20a-5p **	Up	1.12	2.51 × 10^−4^	3.19 × 10^−2^	1044	172
**hsa-miR-4429 **	Down	−1.80	2.77 × 10^−4^	3.33 × 10^−2^	68	20
**hsa-miR-4484 **	Down	−2.13	3.06 × 10^−4^	3.54 × 10^−2^	53	16
**hsa-miR-320d **	Down	−1.54	3.13 × 10^−4^	3.57 × 10^−2^	70	21
**hsa-miR-486-5p**	Up	1.04	3.20 × 10^−4^	3.58 × 10^−2^	54	11
**hsa-miR-6778-5p **	Up	1.82	4.14 × 10^−4^	4.18 × 10^−2^	128	30
**hsa-miR-100-5p**	Down	−0.90	4.73 × 10^−4^	4.61 × 10^−2^	247	80
**hsa-miR-4443**	Down	−1.24	4.75 × 10^−4^	4.61 × 10^−2^	80	13
**hsa-miR-106a-5p**	Up	0.85	4.99 × 10^−4^	4.77 × 10^−2^	673	118
**hsa-miR-3651**	Down	−1.80	5.24 × 10^−4^	4.87 × 10^−2^	30	13

**Table 2:** Relevant highest scoring KEGG pathways modulated by miRNAs differentially expressed in trained (TRA) vs sedentary (SED) vastus lateralis (VL).

KEGG Name	Overlap	*p* Value	Adjusted *p* Value	Differentially Expressed miRNA Target Genes	Number of Target Genes Modulated by miRNAs versus Total Differentially Expressed Genes [7] in the Pathway
Cell cycle	27/124	5.63 × 10^−8^	1.74 × 10^−5^	*RB1;YWHAE;GSK3B;CDKN1A;CUL1;ORC4;CCNB1;CCND2;CCND1;RAD21;ORC2;E2F1;EP300;E2F3;YWHAH;CREBBP;SMAD4;SMAD3;SMC1A;YWHAZ;STAG2;RBL1;TFDP2;ATM;TP53;MAD1L1;ANAPC1*	27/54 (50%)
**AMPK signaling pathway**	21/120	4.05 × 10^−4^	4.16 × 10^−5^	*RAB2A;CPT1A;PRKAA2;STRADB;PPP2R2A;IGF1;PPP2R5C;ADIPOR2;MTOR;CAMKK2;RPTOR;RAB10;CCND1;RPS6KB1;PPP2R5E;SCD;EIF4EBP1;ULK1;PPARG;MAP3K7;PFKP*	21/66 (31%)
TGF-beta signaling pathway	16/90	7.74 × 10^−6^	1.59 × 10^−4^	*CREBBP;SMAD4;SMAD3;CUL1;BMP8B;ACVR1B;SMAD6;RHOA;RGMA;TGFBR2;BMP2;RBL1;RPS6KB1;ID4;EP300;BMPR1A*	16/43 (37%)
**Thyroid hormone signaling pathway**	19/116	3.98 × 10^−6^	1.36 × 10^−4^	*GSK3B;MAP2K1;CREBBP;THRA;NCOA3;ATP2A2;ATP1B3;SLC16A10;ATP1A2;MTOR;MED12;CCND1;TBC1D4;PLCG2;PLCE1;EP300;RAF1;TP53;PFKP*	19/64 (29%)
p53 signaling pathway	13/72	4.58 × 10^−5^	5.23 × 10^−4^	*CDKN1A;PTEN;IGF1;CCNB1;CCND2;CCND1;SESN3;CCNG1;BCL2;FAS;ATM;MDM4;TP53*	13/38 (29%)
**Focal adhesion**	27/199	1.91 × 10^−6^	9.79 × 10^−5^	*GSK3B;FLT1;ROCK2;PTEN;THBS2;ARHGAP35;MYLK3;CRKL;PPP1CB;MAPK8;CCND2;CCND1;FLNA;PAK2;PDGFRA;MAP2K1;PPP1R12A;CAV1;ITGA1;IGF1;RHOA;VEGFA;RAPGEF1;BCL2;GRB2;RAF1;VCL*	27/105 (25%)
FoxO signaling pathway	20/132	7.60 × 10^−6^	1.67 × 10^−4^	*GABARAPL1;MAP2K1;CREBBP;SMAD4;CDKN1A;SMAD3;PRKAA2;PTEN;IGF1;MAPK14;SOD2;TGFBR2;CCNB1;MAPK8;CCND2;CCND1;EP300;GRB2;ATM;RAF1*	20/63 (31%)
**Cellular senescence**	23/160	4.05 × 10^−6^	1.25 × 10^−4^	*RB1;MAP2K1;CDKN1A;SMAD3;TRAF3IP2;PTEN;MAPK14;MTOR;TGFBR2;PPP1CB;CCNB1;CCND2;RBL1;CCND1;RBBP4;EIF4EBP1;E2F1;ATM;E2F3;BTRC;RAF1;TP53;PPID*	23/81 (28%)
Hippo signaling pathway	23/160	4.05 × 10^−6^	1.14 × 10^−4^	*YWHAE;GSK3B;TCF7L2;SMAD4;SMAD3;FZD5;FZD9;BMP8B;PPP2R2A;LIMD1;YWHAZ;AMOT;TGFBR2;PPP1CB;BMP2;CCND2;LATS2;CCND1;DVL3;BTRC;TEAD1;BMPR1A;YWHAH*	23/71 (32%)
*Mitophagy*	12/65	7.12 × 10^−5^	7.31 × 10^−4^	*BECN1;BCL2L13;GABARAPL1;USP15;MAPK8;CSNK2A1;UBB;ATG9A;E2F1;MITF;ULK1;TP53*	12/33 (36%)
*mTOR signaling pathway*	22/152	5.83 × 10^−6^	1.38 × 10^−4^	*GSK3B;MAP2K1;PRKAA2;FZD5;STRADB;FZD9;PTEN;IGF1;RHOA;MTOR;RPTOR;FLCN;SLC7A5;CLIP1;RPS6KB1;EIF4EBP1;DVL3;ULK1;GRB2;RICTOR;RAF1;ATP6V1F*	22/72 (30%)
*Autophagy*	19/128	1.72 × 10^−5^	2.79 × 10^−4^	*BECN1;GABARAPL1;MAP2K1;MTMR3;PRKAA2;ATG9A;PTEN;MTOR;CAMKK2;ERN1;RPTOR;MAPK8;RPS6KB1;LAMP2;ATG2A;BCL2;ULK1;RAF1;MAP3K7*	19/66 (28%)
*Adherens junction*	12/72	1.98 × 10^−4^	1.56 × 10^−3^	*TCF7L2;CREBBP;SMAD4;SMAD3;CSNK2A1;EP300;WASL;MAP3K7;PTPRF;RHOA;VCL;TGFBR2*	12/41 (41%)
*Longevity regulating pathway*	15/102	1.44 × 10^−4^	1.23 × 10^−3^	*PRKAA2;CLPB;IGF1;SOD2;ADIPOR2;MTOR;CAMKK2;RPTOR;SESN3;RPS6KB1;EIF4EBP1;ULK1;PPARG*;*TP53;APPL1*	15/48 (31%)
**Insulin signaling pathway**	18/137	1.44 × 10^−4^	1.20 × 10^−3^	*GSK3B;PYGB;MAP2K1;PRKAA2;PHKA1;PTPRF;HK2;MTOR;CRKL;PPP1CB;RPTOR;MAPK8;RPS6KB1;PRKAR2A;EIF4EBP1;RAPGEF1;GRB2;RAF1*	18/80 (22%)
Wnt signaling pathway	20/158	1.07 × 10^−4^	1.03 × 10^−3^	*GSK3B;TCF7L2;CREBBP;SMAD4;SMAD3;CSNK2A1;FZD5;ROCK2;CUL1;FZD9;PRICKLE1;RHOA;MAPK8;CCND2;CCND1;EP300;DVL3;BTRC;MAP3K7;TP53*	20/73 (27%)
MAPK signaling pathway	32/295	2.71 × 10^−5^	3.63 × 10^−4^	*FLT1;CSF1;MAX;CRKL;ELK4;CACNG6;FGF7;MAPK8;STMN1;FLNA;MAP2K7;PAK2;MAP3K7;MAP4K3;MAP3K2;PDGFRA;MEF2C;MAP2K1;IGF1;IRAK4;MAPK14;VEGFA;TGFBR2;PPM1A;TAOK1;KIT;NF1;FAS;GRB2;RAF1;TP53;MAP3K12*	32/134 (23%)
Neurotrophin signaling pathway	16/119	2.54 × 10^−4^	1.86 × 10^−3^	*YWHAE;GSK3B;MAP2K1;IRAK4;MAPK14;RHOA;CRKL;MAPK8;PLCG2;BCL2;RAPGEF1;GRB2;RAF1;MAP2K7;TP53;NFKBIB*	16/56 (28%)
Sphingolipid signaling pathway	16/119	2.54 × 10^−4^	1.82 × 10^−3^	*MAP2K1;ABCC1;ROCK2;PTEN;PPP2R2A;PPP2R5C;MAPK14;RHOA;GNA13;MAPK8;SPTLC2;PPP2R5E;BCL2;DEGS1;RAF1;TP53*	16/61 (26%)
HIF-1 signaling pathway	14/100	3.98 × 10^−4^	2.56 × 10^−3^	*MAP2K1;CREBBP;CDKN1A;FLT1;CUL2;IGF1;HK2;MTOR;VEGFA;RPS6KB1;EIF4EBP1;PLCG2;BCL2;EP300*	14/54 (26%)
mRNA surveillance pathway	13/91	5.21 × 10^−4^	3.15 × 10^−3^	*HBS1L;SMG1;CPSF7;CPSF2;CSTF2T;PPP2R2A;MSI2;PPP2R5C;MAGOHB;PPP1CB;DDX39B;PPP2R5E;WDR82*	13/37 (35%)
Ferroptosis	7/40	3.15 × 10^−3^	1.62 × 10^−2^	*PRNP;MAP1LC3B;PCBP1;LPCAT3;SLC11A2;ACSL3;TP53*	7/25 (28%)
*Regulation of actin cytoskeleton*	24/214	1.59 × 10^−4^	1.29 × 10^−3^	*NCKAP1;PDGFRA;MAP2K1;PPP1R12A;ROCK2;RDX;ITGA1;LIMK1;WASL;SSH2;RHOA;ARHGAP35;MYLK3;CRKL;PPP1CB;GNA13;FGF7;PIKFYVE;ARPC3;PIP4K2A;RAF1;PAK2;VCL;PFN2*	24/110 (24%)
**Ubiquitin mediated proteolysis**	17/137	4.33 × 10^−4^	2.72 × 10^−3^	*UBE2H;FBXW8;CUL5;UBE2B;FBXW7;MGRN1;CUL3;CUL2;CUL1;HUWE1;UBE4B;UBE2S;UBE2Q2;UBE2N;UBA2;BTRC;ANAPC1*	17/73 (23%)

KEGG pathways are listed in descending order of the combined score, which is described as c = log(p) * z, where c = the combined score, *p* = Fisher exact test *p*-value, and z = z-score for deviation from expected rank [36]. Kyoto Encyclopedia of Genes and Genomes (KEGG, Release 91.0, 1 July 2019) was used. KEGG pathways in bold are also found within the highest scoring ones characterizing the set of differentially expressed mRNAs, KEGG pathways in italic are additional ones characterizing differentially regulated pathways or processes [7]. Overlap: No. of differentially expressed miRNA target genes that overlap total genes of the pathway.

**Table 3:** LncRNAs differentially expressed in trained (TRA) vs sedentary (SED) vastus lateralis (VL) and their differentially expressed mRNA and miRNA targets.

Ensembl gene ID	Gene Symbol	log2 FC	*p* Value	Adj *p* Value	Validated Targets That Are Differentially Expressed [7]
ENSG00000233016	*SNHG7*	−1.42	0.000000	0.000007	*GALNT7* (−0.91); *FAIM2* (−0.72)
ENSG00000177410	*ZFAS1*	−2.29	0.000000	0.000009	*KLF2* (−3.06); *ZEB1* (0.87); *TJP1* (0.61)
ENSG00000234741	*GAS5*	−1.93	0.000000	0.000016	*ANXA2* (−2.6); *CCND1* (−1.23); *E2F1* (−1.12); *KLF2* (−3.06); *MMP2* (−2.33); *PTEN* (−0.48); *SMAD3* (1.12); *TP53* (−1.12); *VEGFA* (2.60); *VIM* (−3.32); *EIF4E* (0.64); *IGF1R* (0.38); hsa−miR106a−5p (0.85)
ENSG00000224078	*SNHG14*	1.97	0.000002	0.000084	*WASL* (0.51)
ENSG00000224259	*RP11-48O20.4* (*LINC01133*)	−4.41	0.000000	0.000016	*KLF2* (−3.06)
ENSG00000163597	*SNHG16*	0.71	0.000001	0.000048	*FBXW7* (1.22)
ENSG00000224189	*HOXD-AS1* (*HAGLR*)	−2.25	0.000003	0.000108	*RUNX3* (−1.64)
ENSG00000197989	*SNHG12*	−1.51	0.000004	0.000149	*AMOT* (2.00); *NOTCH2* (−0.59); hsa−miR−181a (1.90)
ENSG00000229847	*EMX2OS*	−1.46	0.000038	0.000695	*EMX2* (−1.87)
ENSG00000233429	*HOTAIRM1*	−1.40	0.000018	0.000413	hsa−miR20a−5p (1.12)
ENSG00000227372	*TP73-AS1*	0.50	0.000284	0.003178	*BDH2* (−0.72)
ENSG00000232956	*SNHG15*	−0.91	0.000355	0.003793	*KLF2* (−3.06); *MMP2* (−2.33); *MMP9* (−3.90)
ENSG00000222041	*LINC00152* (*CYTOR*)	−1.95	0.000577	0.005579	*CYTOR* (−2.33); *BCL2* (0.82); *CDKN1A* (−2.56); *PIK3CB* (0.85); *VIM* (−3.32); *FN1* (−1.73)
ENSG00000255717	*SNHG1*	−0.57	0.001889	0.014430	*TP53* (−1.12); *VIM* (−3.32)
ENSG00000250451	*HOXC-AS1*	−0.86	0.002378	0.017336	*HOXC6* (−0.55)
ENSG00000251562	*MALAT1*	0.67	0.006092	0.037387	*PCNA* (−0.56); *CSF1* (−1.94); *CA2* (2.39); *GPC6* (−2.34); *BMPER* (−1.10); *CA2* (2.39); *GPC6* (−2.34); *CSF1* (−1.94); *LPAR1* (−2.53); *COL6A1* (−0.99); *MCAM* (−1.18); *STC1* (−2.51); *NNMT* (−3.09); *CPM* (−2.03); *CASP9* (0.62); *CCL2* (−2.68); *CCND1* (−1.23); *CDKN1A* (−2.56); *MMP2* (−2.33); *ZEB1* (0.87); *SNAI2* (−1.31); *MAP2K1* (0.87); *MAPK1* (0.67); *MAPK14* (0.52); *MAPK3* (−1.12); *MAPK8* (0.58); *MAPK9* (0.96); *SFRP1* (−2.97); *CLDN5* (−1.99); *OCLN* (−1.32); *CTHRC1* (−2.36); *PTBP3* (−0.63); *MMP14* (−2.30); *PXN* (1.26); *PIK3CB* (0.85); *SNCA* (−3.25); *TP53* (−1.12); *ROBO1* (−0.88); *PRKCE* (0.67); *CASP3* (−0.42); *CDH5* (0.59); *ZEB2* (−0.67); *FN1* (−1.73); *TJP1* (0.61); *RAP1B* (−0.58)

The gene name of lncRNAs is that assigned in the Ensembl Release GRCh37.72. When it differs in the more recent Release GRCh38.99, the new name is reported in parentheses. Log2 Fc of differentially expressed targets is shown in parentheses next to the gene name.

## Data Availability

All data are reported in Tables S1 and S4. Data obtained from the Affimetrix arrays and the NGS analysis have been deposited in GEO (GSE165633 https://www.ncbi.nlm.nih.gov/geo/query/acc.cgi?acc=GSE165633).

## Supplementary Materials

Supplementary Materials can be found at https://www.mdpi.com/1422-0067/22/4/1539/s1. Table S1: All sequences identified by the Affimetrix array. Metric shows the results of TRA/SED ratio. Table S2: Differentially expressed targets of miRNAs; Table S3: 50 highest-scoring KEGG pathways identified by analyzing a panel of 989 validated targets of differentially expressed miRNAs. The panel included only mRNAs which were at the same time differentially expressed in TRA and in SED and with opposite trends to their cognate miRNAs. The Kyoto Encyclopedia of Genes and Genomes (KEGG, Release 91.0, July 1, 2019) was used. KEGG pathways are listed in descending order of the combined score, which is defined as c = log(p) * z, where c = the combined score, *p* = Fisher exact test *p*-value, and z = z-score for deviation from expected rank [36]. Overlap: no. of genes/ total genes in the pathway. Table S4: All lncRNA sequences identified by Next Generation Sequencing. Metric shows the results of TRA/SED ratio.

## Abbreviations

SED	sedentary
TRA	trained
ET	endurance training
RT	resistance training
KEGG	Kyoto Encyclopedia of Genes and Genomes
NGS	Next Generation Sequencing
miRNA	microRNA
lncRNA	long non-coding RNA
cpm	counts per million
